# A cross-sectional study of factors associated with carotid atherosclerosis

**DOI:** 10.3389/fphys.2024.1434173

**Published:** 2024-10-18

**Authors:** Guokui Dai, Xiangsheng Cai, Chuanjiang Ye, Yuzhen Zhang, Ruoping Guan

**Affiliations:** ^1^ Clinical Laboratory, Guangzhou Cadre and Talent Health Management Center, Guangzhou, China; ^2^ Department of Ultrasound, Guangzhou Cadre and Talent Health Management Center, Guangzhou, China

**Keywords:** carotid atherosclerosis, influencing factors, new insulin resistance index, cross-sectional study, multivariate logistic regression analysis

## Abstract

**Objective:**

The aim of this work was to study the relationship between carotid atherosclerosis (CAS) and several indexes and provide a basis for the prevention and treatment of cardiovascular and cerebrovascular diseases.

**Methods:**

There were 11,028 adults who underwent physical examination at the Guangzhou Cadre and Talent Health Management Center from January 2023 to December 2023 and were selected as research subjects. Retrospective analysis was used to understand the carotid atherosclerosis of the examined population and analyze its relationship with sex, age, blood pressure, blood glucose, blood lipids, renal function, 25-hydroxyvitamin D, neutrophil to lymphocyte count ratio (NLR), platelet to lymphocyte count ratio (PLR), systemic immune inflammation index (SII), monocyte count to high-density lipoprotein cholesterol ratio (MHR), triglyceride glucose body mass index (TyG-BMI), insulin resistance metabolic index (METS-IR), and other indicators.

**Results:**

Among 11,028 subjects, the detection rate of carotid atherosclerotic thickening (CAT) was 12.00% and carotid atherosclerotic plaque (CAP) was 25.11%. The CAT and CAP detection rates in men were 13.32% and 28.78%, respectively, which were higher than the CAT detection rate of 8.28% and CAP detection rate of 14.80% in women, and the differences were statistically significant (both *p* < 0.001). Multivariate logistic regression analysis using TyG-BMI and METS-IR as two indicators was modeled separately, and the results showed that CAS was associated with men, increasing age, and systolic blood pressure. The area under the curve (AUC) was analyzed using the subject’s work characteristic (ROC) curve in the descending order of METS-IR, TyG-BMI, and MHR. The combination of the three indexes of sex, age, and METS-IR predicted atherosclerosis with the highest AUC values.

**Conclusion:**

Carotid atherosclerosis is highly prevalent in men. Elevation of systolic blood pressure, fasting glucose, MHR, and TyG-BMI (or METS-IR) with age are independent influences on carotid atherosclerosis. The three indexes of MHR, TyG-BMI, and METS-IR, respectively, in combination with sex and age, can be used as a new and effective index to predict CAS.

## Introduction

Atherosclerosis (AS) is a chronic inflammatory disease caused by abnormal lipid metabolism (Maxfield et al., 2023), oxidative stress (Ménégaut et al., 2023), and endothelial damage (Yuan et al., 2022) that occurs throughout the development of as the disease. AS is a major cause of coronary heart disease, peripheral vascular disease, and cerebral infarction (Su et al., 2021). As AS progresses, plaque rupture can cause cardiovascular and cerebrovascular emergencies, seriously jeopardizing human life and health.

Carotid atherosclerosis (CAS) occurs most often in adult men over 40 years of age and is associated with a variety of factors such as smoking, hypertension, and diabetes (Fang et al., 2024; Đermanović Dobrota et al., 2024; Yu et al., 2024). Clinically, CAS is mainly diagnosed using imaging, which requires experienced physicians and may be missed in the early stages (Mishra et al., 2022). Some literature studies reported the relationship between early atherosclerosis and insulin resistance index (Scott et al., 2024). The use of baseline data plus blood biochemical indices to predict CAS in its early stages shows potential to achieve early recognition of CAS. Therefore, it is important to find new indicators that can be used to predict CAS.

In recent years, there have been many reports on derived calculation indicators such as blood cell count and clinical biochemistry, including monocyte count to high-density lipoprotein cholesterol ratio (MHR), triglyceride glucose body mass index (TyG-BMI), and insulin resistance metabolic score (METS-IR). As a new inflammatory response marker and insulin resistance index, they have been applied in various diseases along with traditional indicators such as HOMA-IR and waist circumference (Zhang et al., 2023; Zhao et al., 2022; Feng et al., 2024; Cho et al., 2020). Another study confirmed that elevated levels of TyG-BMI are significantly and positively associated with stroke risk in middle-aged and elderly populations (Shao et al., 2024). However, no relevant study on the relationship between CAS, TyG-BMI, and METS-IR has been seen. In this study, the relationship between CAS and several indicators was analyzed by using 11,028 medical checkups in the health checkups at the Guangzhou Cadre and Talent Health Management Center as the study subjects so that the relationship between CAS and several indexes could be analyzed to provide the basis for the prevention and treatment of CAS.

## Materials and methods

### Subjects

**FIGURE 1:**
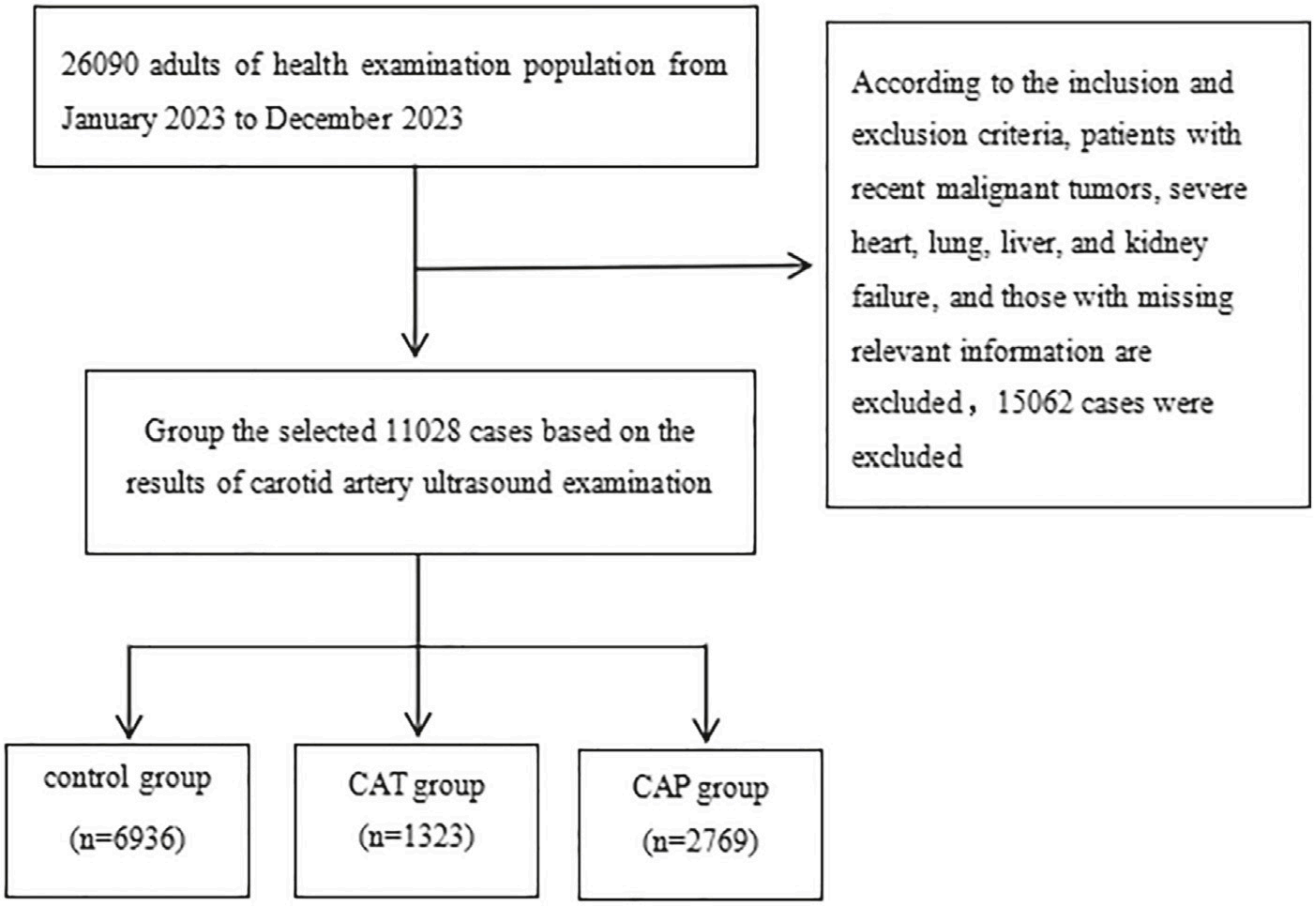
Flowchart for recruiting participants.

The study population was selected for a cross-sectional survey study, selecting adults who had health checkups at the Guangzhou Cadre and Talent Health Management Center from January 2023 to December 2023. Inclusion criteria: complete carotid ultrasound and laboratory examination data; exclusion criteria: ① patients who had recently suffered from malignant tumors or severe heart, lung, liver, or kidney failure; ② pregnant and lactating women; and ③those with missing examination data. People taking hypoglycemic and antihypertensive drugs and those suffering from metabolic diseases were not excluded, and 11,028 cases were finally included in the study, with a mean age of (51.44 ± 8.09) years, of which 8,130 (73.72%) were men and 2,898 (26.28%) were women (Figure 1). According to the results of carotid ultrasound examination, they were divided into plaque , hyperplasia, and control groups. All participants signed an informed consent form, and the study was approved by the Ethics Committee of the Guangzhou Cadre and Talent Health Management Center (approval number: JGZX-2024–13).

### Physical examination

Physical examination was performed by uniformly trained healthcare personnel. Height (m) and body mass (kg) were measured using a calibrated ultrasonic height and weight meter, and the body mass index (BMI = kg/m2) was calculated. Systolic blood pressure (SBP) and diastolic blood pressure (DBP) were measured using the OMRON (HBP-9020) automatic electronic sphygmomanometer; all the medical examiners sat still for 10 min before the measurement, and the brachial artery of the right upper limb was taken for the measurement, which was measured three times, and the average value was taken. The unit of blood pressure was mmHg.

### Echography

All research subjects were subjected to carotid artery ultrasound examination by trained and certified ultrasound physicians using a color Doppler ultrasound diagnostic instrument (Siemens Sequoia) equipped with a probe model of 10L4 and a frequency of 10 MHz. All subjects were evaluated by carotid ultrasound at the same time by two experienced physicians, and the diagnosis was clear if the results were consistent. If the results were inconsistent, the final diagnosis was made by a superior physician. Subjects with carotid intima-media thickness IMT <1.0 mm and smooth intima-media were included in the control group; subjects with carotid atherosclerotic thickening (CAT) at 1.0 mm ≤ IMT <1.5 mm were included in the thickening group; and subjects with carotid atherosclerotic plaque (CAP) at IMT ≥1.5 mm were included in the plaque group. CAS included CAT and CAP.

### Laboratory examinations

Fasting venous blood was collected from the subjects, serum was separated, and 25-hydroxyvitamin D (25(OH)D) was measured using a Roche cobas 801 electrochemiluminescence meter. Fasting blood glucose (FPG), triacylglycerol (TG), total cholesterol (TCHO), high-density lipoprotein cholesterol (HDL-c), low-density lipoprotein cholesterol (LDL-c), urea (Urea), blood creatinine (Scr), and blood uric acid (BUA) were detected using a Canon TBA FX-8 biochemistry meter. In addition, 2 mL of EDTA-K2 anticoagulant whole blood was collected, and blood cell counts were performed using a Myriad BC-6800 hematology analyzer. NLR, PLR, SII, MHR, TyG-BMI, and METS-IR were calculated as follows.

NLR, neutrophil count/lymphocyte count;
PLR, platelet count/lymphocyte count;
SII, (neutrophil count × platelet count)/lymphocyte count;
MHR, monocyte count/HDL-C;
TyG-BMI = ln [TG (mg/dL) × FPG (mg/dL)/2] × BMI
METS-IR = ln [2 × FPG (mg/dL)+TG (mg/dL)] × BMI/ln [HDL-C (mg/dL)].

### Statistical processing

Analysis was performed using SPSS 25.0 statistical software. Measurement information was expressed as the normality test and the chi-squared test, the t-test (or t′test) was used for intergroup comparisons, and the Wilcoxon signed rank-sum test and the rank-sum test for multiple-sample comparisons (Kruskal–Wallis method) were used for non-normal paired information. Multivariate logistic regression analysis (forward step method) was used for arteriosclerosis exposure factor analysis, and odds ratios (ors) and 95% confidence intervals (95% CI) were calculated to analyze the relationship between cervical arteriosclerosis and exposure factors. The χ^2^chi-squared test was used for count data, and *p* < 0.05 indicated a statistically significant difference.

## Results

### Clinical data on the study population

Among the 11,028 subjects, the detection rate of carotid atherosclerotic thickening (CAT) was 12.00% (1323/11,028) and carotid atherosclerotic plaque (CAP) was 25.11% (2769/11,028). The detection rates of CAT and CAP in male subjects were 13.32% (1083/8130) and 28.78% (2340/8130), respectively, both higher than those of CAT 8.28% (240/2898) and CAP 14.80% (429/2898) in female subjects, and the differences were statistically significant (both *p* < 0.001).

In male subjects, the CAT detection rate was similar in those aged 65 years and older compared with those less than 65 years, with no statistical difference (*p* > 0.05), while the CAP detection rate was significantly higher, with a statistically significant difference (*p* < 0.001). Among female subjects, CAT and CAP detection rates were elevated in those aged 65 years and above compared with those aged below 65 years, with statistically significant differences (both *p* < 0.001), and were close to those of men in the same age group (both *p* > 0.05).

There were differences in sex ratio, age, BMI, SBP, DBP, 25(OH)D, FPG, TG, TCHO, HDL-C, LDL-c, Urea, BUA, Scr, NLR, PLR, MHR, TyG-BMI, and METS-IR among the three groups, and the differences were statistically significant (all *p* < 0.01), while no statistical difference was seen in the SII results between the three groups (*p* > 0.05). See Table 1.

**TABLE 1:** Clinical data on the study population [n (%), $\bar{x}\pm s$ ].

	Control group (n = 6,936)	CAT group (n = 1,323)	CAP group (n = 2,769)	F/χ2	P
Male					
<65 years	4588/7645 (60.01)	1008/7645 (13.19)	2049/7645 (26.80)	χ*2 =* 275.5576	<0.001
≥65 years	119/485 (24.54)	75/485 (15.46)	291/485 (60.00)		
Female					
<65 years	2181/2727 (79.98)	213/2727 (7.81)	333/2727 (12.21)	χ*2 =* 278.5006	<0.001
≥65 years	48/171 (28.07)	27/171 (15.79)^a^	96/171 (56.14)^a^		
Age (years)	49.22 ± 6.83	53.76 ± 7.63	55.89 ± 9.03	F = 1170.159	<0.001
BMI (kg/m^2^)	24.36 ± 3.05	25.00 ± 3.12	25.06 ± 2.98	F = 117.051	<0.001
SBP (mmHg)	119.67 ± 14.85	124.18 ± 14.86	126.74 ± 15.68	F = 367.933	<0.001
DBP (mmHg)	71.78 ± 10.91	74.13 ± 10.71	75.09 ± 10.82	F = 164.171	<0.001
25(OH)D (ng/mL)	22.05 ± 6.79	23.33 ± 6.74	23.72 ± 7.31	F = 122.028	<0.001
FPG (mmol/L)	5.31 ± 0.92	5.50 ± 1.15	5.71 ± 1.39	F = 202.978	<0.001
TG [M(Q1, Q3), mmol/L]	1.22 (0.87, 1.80)	1.34 (0.96, 1.91)	1.39 (0.99, 2.02)	F = 166.369	<0.001
TCHO (mmol/L)	5.16 ± 0.92	5.25 ± 0.98	5.21 ± 1.06	F = 10.759	<0.001
HDL-C (mmol/L)	1.48 ± 0.35	1.42 ± 0.34	1.40 ± 0.32	F = 107.601	<0.001
LDL-C (mmol/L)	2.94 ± 0.78	3.07 ± 0.83	3.01 ± 0.89	F = 24.485	<0.001
Urea (mmol/L)	5.24 ± 1.28	5.51 ± 1.42	5.60 ± 1.49	F = 137.377	<0.001
Scr (umol/L)	78.14 ± 23.87	82.33 ± 40.41	82.60 ± 30.13	F = 75.041	<0.001
BUA (umol/L)	405.8 ± 102.7	423.6 ± 99.9	426.1 ± 98.1	F = 125.183	<0.001
NLR	1.86 ± 0.78	1.89 ± 0.82	1.91 ± 0.90	F = 4.729	0.009
PLR	135.73 ± 49.15	132.68 ± 47.80	129.14 ± 43.84	F = 19.228	<0.001
SⅡ	457.5 ± 241.3	458.2 ± 256.3	458.7 ± 256.4	F = 0.024	0.977
MHR	0.26 ± 0.12	0.28 ± 0.12	0.30 ± 0.14	F = 82.814	<0.001
TyG-BMI	91.33 ± 15.38	94.72 ± 15.33	95.93 ± 15.05	F = 100.138	<0.001
METS-IR	35.20 ± 6.60	36.74 ± 6.67	37.22 ± 6.47	F = 106.155	<0.001

1 mmHg = 0.133 kPa.

### Multivariate logistic regression analysis of CAS and related indicators

The variances were multicollinearity, and the variance expansion factor (VIF) was calculated. The VIF of TyG-BMI and METS-IR were both > GT. 10, which had collinearity. The Pearson chi-squared test showed that the goodness of fit of Model 1 was 0.877 and that of Model 2 was 0.874.

Model 1 results showed that CAT was associated with male sex (OR = 1.822, 95% CI: 1.546–2.147, *p* < 0.001), age (OR = 1.086, 95% CI: 1.077–1.095, *p* < 0.001), systolic blood pressure (OR = 1.005, 95% CI: 1.000–1.009, *p* = 0.036), and TyG-BMI (OR = 1.007, 95% CI: 1.002–1.012, *p* = 0.005). CAP was independently associated with male sex (OR = 1.993, 95% CI: 1.744–2.278, *p* < 0.001), age (OR = 1.112, 95% CI: 1.104–1.120, *p* < 0.001), systolic blood pressure (OR = 1.009, 95% CI: 1.006–1.013, *p* < 0.001), fasting blood glucose (OR = 1.095, 95% CI: 1.048–1.143, *p* < 0.001), MHR (OR = 2.903, 95% CI: 1.942–4.340, *p* < 0.001), and TyG-BMI (OR = 1.006, 95% CI: 1.002–1.010, *p* = 0.001). See Table 2.

**TABLE 2:** Multivariate logistic regression analysis of variables related to CAS.

Variable	β	S.E.	Wald χ2	*p*-value	OR (95% CI)
CAT group compared with the control group					
Male	0.600	0.084	51.372	<0.001	1.822 (1.546, 2.147)
Age	0.083	0.004	378.003	<0.001	1.086 (1.077, 1.095)
SBP	0.005	0.002	4.382	0.036	1.005 (1.000, 1.009)
FPG	0.003	0.031	0.011	0.916	1.003 (0.943, 1.067)
MHR	0.162	0.270	0.360	0.548	1.176 (0.692, 1.999)
TyG-BMI	0.007	0.002	7.933	0.005	1.007 (1.002, 1.012)
CAP group compared with the control group					
Male	0.690	0.068	102.186	<0.001	1.993 (1.744, 2.278)
Age	0.106	0.004	912.494	<0.001	1.112 (1.104, 1.120)
SBP	0.009	0.002	27.968	<0.001	1.009 (1.006, 1.013)
FPG	0.091	0.022	16.645	<0.001	1.095 (1.048, 1.143)
MHR	1.066	0.205	26.982	<0.001	2.903 (1.942, 4.340)
TyG-BMI	0.006	0.002	10.431	0.001	1.006 (1.002, 1.010)

Abbreviations: OR, odds ratio; CI, confidence interval.

Model 2 results showed that CAT was independently associated with male sex (OR = 1.791, 95% CI: 1.518–2.112, *p* < 0.001), age (OR = 1.087, 95% CI: 1.078–1.096, *p* < 0.001), systolic blood pressure (OR = 1.005, 95% CI: 1.000–1.009, *p* = 0.036), and METS-IR (OR = 1.020, 95% CI: 1.008–1.032, *p* = 0.001). CAP was associated with male sex (OR = 1.980, 95% CI: 1.731–2.265, *p* < 0.001), age (OR = 1.112, 95% CI: 1.104–1.120, *p* < 0.001), systolic blood pressure (OR = 1.009, 95% CI: 1.006–1.013, *p* < 0.001), fasting glucose (OR = 1.097, 95% CI: 1.050–1.145, *p* < 0.001), MHR (OR = 2.664, 95% CI: 1.747–4.063, and *p* < 0.001), and METS-IR (OR = 1.015, 95% CI: 1.006–1.024, *p* = 0.002). See Table 3.

**TABLE 3:** Multivariate logistic regression analysis of variables related to CAS.

Variable	Β	S.E.	Wald χ2	*p*-value	OR (95% CI)
CAT group compared with the control group					
Male	0.583	0.084	47.814	<0.001	1.791 (1.518, 2.112)
Age	0.083	0.004	381.089	<0.001	1.087 (1.078, 1.096)
SBP	0.005	0.002	4.397	0.036	1.005 (1.000, 1.009)
FPG	0.000	0.031	0.000	0.989	1.000 (0.941, 1.064)
MHR	−0.005	0.285	0.000	0.987	0.995 (0.570, 1.739)
METS-IR	0.020	0.006	11.262	0.001	1.020 (1.008, 1.032)
CAP group compared with the control group					
Male	0.683	0.069	98.977	<0.001	1.980 (1.731, 2.265)
Age	0.106	0.004	911.823	<0.001	1.112 (1.104, 1.120)
SBP	0.009	0.002	29.502	<0.001	1.009 (1.006, 1.013)
FPG	0.092	0.022	17.480	<0.001	1.097 (1.050, 1.145)
MHR	0.980	0.215	20.713	<0.001	2.664 (1.747, 4.063)
METS-IR	0.015	0.005	9.940	0.002	1.015 (1.006, 1.024)

Abbreviations: OR, odds ratio; CI, confidence interval.

### Three derived computational indicators predict carotid atherosclerosis

**FIGURE 2:**
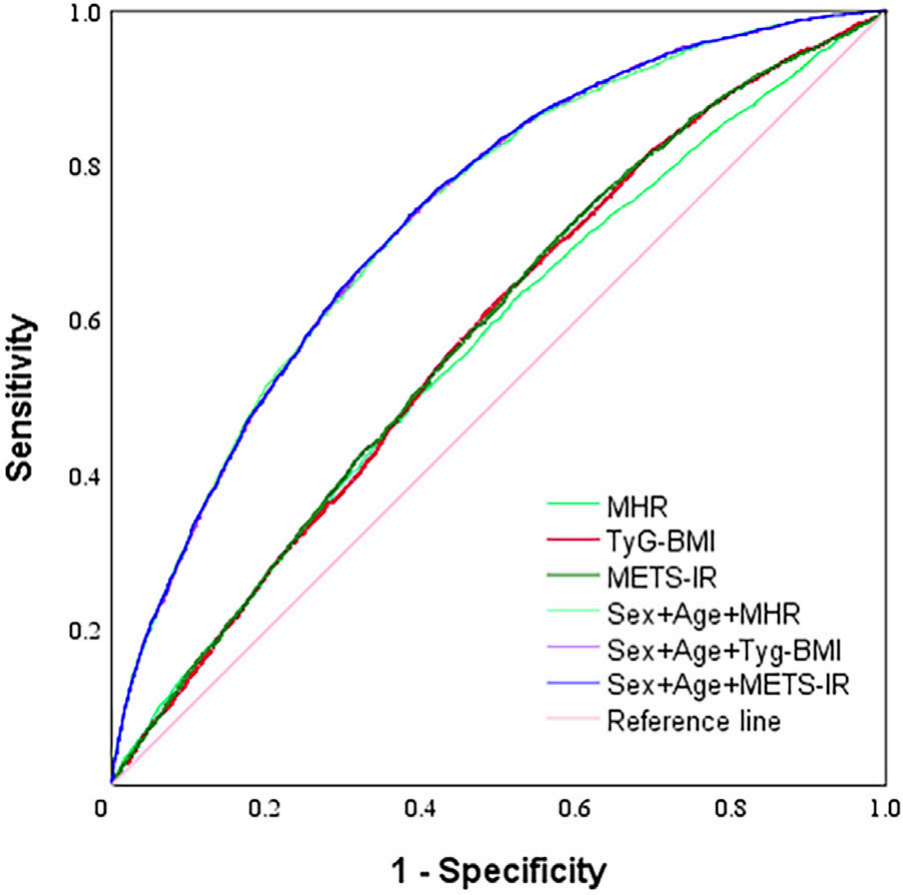
ROC analyses of three derived calculation indexes on carotid atherosclerosis.

The value of each index in predicting CAS was analyzed by using the subject’s work characteristics (ROC) curve, and the area under the curve (AUC) was, in descending order, METS-IR (0.585), TyG-BMI (0.582), and MHR (0.569), which were all statistically different (*p* < 0.001). The AUCs of the three indicators (METS-IR, TyG-BMI, and MHR) combined with gender and age to predict CAS were 0.738, 0.737, and 0.736, respectively, which were higher than the AUCs of the individual indicators, and the differences were statistically significant (all *p* < 0.001), with the METS-IR combining gender and age to predict CAS having the largest AUC value (Table 4 and Figure 2).

**TABLE 4:** Prediction results of three derived calculation items for carotid atherosclerosis.

	Best tangent value	Specificity	Sensitivity	Youden′s index	AUC (95% CI)	Standard error	P
MHR	0.234	0.469	0.638	0.107	0.569 (0.558–0.580)	0.006	<0.001
TyG-BMI	83.387	0.322	0.800	0.122	0.582 (0.571–0.593)	0.006	<0.001
METS-IR	33.017	0.399	0.733	0.132	0.585 (0.574–0.596)	0.006	<0.001
Sex + age + MHR		0.598	0.751	0.349	0.736 (0.727–0.745)	0.005	<0.001
Sex + age + TyG-BMI		0.590	0.759	0.349	0.737 (0.728–0.747)	0.005	<0.001
Sex + age + METS-IR		0.580	0.771	0.351	0.738 (0.729–0.747)	0.005	<0.001

## Discussion

CAS plaques are a common cause of ischemic stroke. Conventional ultrasound is non-invasive, convenient, and accurate in assessing CAS (Sebastian et al., 2024). CAS develops in response to multiple factors, and it is consistent with health economics to look for relevant contributing factors and to intervene effectively in the early stages . Therefore, it is particularly important to use more detection methods as early screening or auxiliary diagnosis in the early stage of CAS, and the combination of baseline data and clinical biochemical indicators with ultrasonic evaluation, is a better choice.

Aging is a key risk factor for atherosclerosis (Piechocki et al., 2024), and thickening of the carotid intima-media is a natural physiologic process with age. In our study population, the detection rate of CAT was close to that reported by (Ma et al., 2022) but lower than the results of a large-scale national epidemiologic survey (Fu et al., 2024), while the detection rate of carotid plaques was close to that reported in China (Ma et al., 2022; Fu et al., 2024). The reason for the difference is considered to be caused by geography, age, occupation and sampling error. The subjects of this study were mostly civil servants, with relatively high health literacy and slightly lower prevalence of obesity, diabetes, and other diseases, which is the reason for the lower detection rate of CAS. It is noteworthy that the detection rates of CAT and CAP in women were lower than those in men of the same age group before 65 years of age, but the detection rates of CAT and CAP in women increased significantly after 65 years of age and were close to those of CAT and CAP in men of the same age group, which may be attributed to the fact that estrogen has a vasoprotective effect on the vasculature of the female population before menopause; however, estrogen levels drop after menopause, and it is possible that estrogen has a protective effect on blood vessels in the female population before menopause, but after menopause, the estrogen level decreases and its receptor expression or function is abnormal, thus losing its protective effect on arteries (Davezac et al., 2021).

Regression results showed that CAT was associated with sex, age, and systolic blood pressure, which was consistent with previous reports (Hou et al., 2018). In addition, CAP was independently associated with sex, age, systolic blood pressure, and fasting blood glucose, consistent with traditional reports (Fu et al., 2024). The study did not observe a correlation between CAT and fasting blood glucose. Considering that the study population was mainly a healthy medical checkup population, the fasting blood glucose level was mostly within the reference interval, and fluctuation of blood glucose within the reference interval was not enough to cause CAT, which also deserves further study.

In recent years, with the application of big data and intelligent technology, some new inflammatory response markers and insulin resistance index indicators calculated by conventional items have been reported more often, and the combination of several indicators can sensitively reflect the small changes in disease status, expanding the application scope of common items, such as NLR and PLR, which are used in the assessment and prognosis of cardiovascular and cerebrovascular diseases (Feng et al., 2024; Bao et al., 2020). MHR is used for the prediction of metabolic diseases (Marra et al., 2024), which is significantly associated with all-cause and cardiovascular mortality in the general population (Jiang et al., 2022), and could be used as a new prognostic biomarker for stroke (Gkantzios et al., 2023). Research has shown (Jiang et al., 2022) that soluble pro-inflammatory factors secreted by neutrophils promote damage to the vascular endothelium, allowing accelerated platelet aggregation and adhesion and promoting plaque development, and that they interact with plaque growth. Lymphocytes are indicators of acquired adaptive immunity and are reduced in response to inflammation. Monocyte recruitment is present during atherosclerosis, and monocytes migrate and differentiate into macrophages, which become macrophage foam cells after phagocytosis of lipids. Among the novel inflammatory markers and insulin resistance index indicators in this study, MHR was positively correlated with CAP, TyG-BMI and METS-IR were positively correlated with carotid atherosclerosis, and no correlation was found between carotid atherosclerosis and NLR, PLR, and SII, which is different from previous reports (Song et al., 2024). It may be related to differences in the subjects under study including the degree of inflammatory response, number and nature of plaques, and the number of macrophages.

TyG-BMI is a derivative of the three items, and there is a high correlation with the METS-IR index, both of which can comprehensively reflect the metabolic state of the body, and the two indexes show high accuracy in predicting diabetes mellitus (Shangguan et al., 2024), which is a reliable alternative index for insulin resistance. Insulin resistance is a common pathophysiological mechanism for a variety of metabolic diseases including diabetes mellitus, hypertension, and dyslipidemia. Insulin resistance can lead to elevated blood glucose, which promotes the production of oxygen free radicals, activates the MAPK/NF-kB transduction pathway, and ultimately leads to vascular endothelial cell injury. Cross-sectional studies have reported a positive correlation between TyG index and CAS (Chen et al., 2023). Visceral adipose tissue in people with elevated BMI, which also releases pro-inflammatory cytokines, contributes to increased atherosclerosis risk, and cohort studies have shown that BMI in childhood and adolescence is positively associated with carotid intima-media thickness in adulthood (Ramezankhani et al., 2023), and BMI increases the risk of atherosclerosis (Liu J. et al., 2023). Findings from a large population-based study in East Asia suggest that TyG-BMI is positively associated with both prehypertension and hypertension (Huang et al., 2023). It has also been shown that TyG-BMI has a higher impact and predictive value in predicting return to normoglycemia or progression to diabetes in prediabetes compared to TyG and BMI (Yang et al., 2024). In our study, there was a positive correlation between TyG-BMI and carotid atherosclerosis, and a correlation with CAT and CAP development, which, to the best of our knowledge, is the first report of a positive correlation between TyG-BMI and CAS. Another study found that elevated TyG-BMI was significantly and positively associated with the risk of stroke in the middle-aged and elderly Chinese population (Shao et al., 2024), which is consistent with our study.

In the present study, there was also a positive correlation between METS-IR and CAS and a correlation with CAT and CAP. To the best of our knowledge, this is the first report of a positive association between METS-IR and CAS. Dyslipidemia is a major risk factor for atherosclerosis, and HDL-C is generally recognized as a protective factor for vascular health. The METS-IR index increased the HDL-C parameter compared with TyG-BMI, but in the present study, the efficacy of METS-IR to improve the CAS test was more limited when compared with TyG-BMI.

There are few reports on the use of MHR, TyG-BMI, and METS-IR indicators to predict CAS; there are also almost no reports on the use of TyG-BMI and METS-IR indicators to predict CAS. In this study, the combination of the three indexes with sex and age can better predict CAS lesions, which has a certain application value. In the present study, the combination of sex, age, and METS-IR had the highest AUC value and the best effect in predicting CAS. To the best of our knowledge, this is the first report of the combination of sex, age, and METS-IR for CAS. TyG-BMI, METS-IR, and MHR indicators are easy to obtain and may be important contributing factors to atherosclerosis, which deserves further study. Meanwhile, TyG-BMI, METS-IR, and MHR indexes may also be potentially valuable for risk assessment and efficacy monitoring of atherosclerosis high-risk groups.

It has been reported that vitamin D levels had no significant effect on carotid intima-media thickness (Rashid, 2024), which is similar to the conclusion of the present study that there was no correlation between CAS and vitamin D levels, and it has also been reported that there was a tendency for VitD levels to decrease in people with increased carotid intima-media thickness (Liu CY. et al., 2023). In addition, the difference in the conclusions may be due to the fact that the people who were taking vitamin D were not excluded from the present study. Vitamin D is not only necessary for maintaining bone health but also plays an important role in immune regulation, lipid metabolism, anti-inflammation, and apoptosis, which affects thrombosis and vascular health. Of interest, vitamin D levels have an impact on the levels of several inflammatory markers (Tang et al., 2023; Elgormus et al., 2023). In this study, CAS did not correlate with the levels of some inflammatory marker indicators, and whether it is associated with vitamin D supplementation and other nutrients deserves further investigation.

The shortcomings of this study are that despite the relatively large number of cases studied, it is a single-center cross-sectional study; hence, the distribution of certain populations may vary, and individual questionnaire responses might be biased. In addition, drugs may affect the results of the study, and this study did not group statistics on the medication taken by the medical examination population. Therefore, it would be more convincing to choose multi-center studies, exclude influencing factors, and conduct closer follow-up studies in future work.

In conclusion, CAT rises with age, is highly prevalent in men, and is independently associated with elevated systolic blood pressure, TyG-BMI, and METS-IR. CAP also rises with age, is highly prevalent in men, is independently associated with elevated systolic blood pressure, fasting glucose, MHR, TyG-BMI, and METS-IR, and should be closely monitored and managed by controlled health interventions. MHR, TyG-BMI, and METS-IR, combined with sex and age, have a certain predictive ability for CAS, and the combination of sex, age, and METS-IR has the highest AUC and the best effect in predicting atherosclerosis. These studies provide useful support for early screening and diagnosis of CAS in clinical practice.

## Data Availability

The original contributions presented in the study are included in the article/Supplementary Material; further inquiries can be directed to the corresponding authors.
